# Supplementary material for: Cell type-specific driver lines targeting the Drosophila central complex and their use to investigate neuropeptide expression and sleep regulation
Source: bioRxiv. 2024 Oct 22:2024.10.21.619448. Preprint. [Version 1] doi: 10.1101/2024.10.21.619448 (PMC11526984; doi:10.1101/2024.10.21.619448)
Supplement: Supplementary file 1 [file NIHPP2024.10.21.619448V1-supplement-1.pdf]

bioRxiv preprint doi: https://doi.org/10.1101/2024.10.21.619448; this version posted October 22, 2024. The copyright holder for this preprint (which was not certified by peer review) is the author/funder, who has granted bioRxiv a license to display the preprint in perpetuity. It is made available under aCC-BY 4.0 International license.

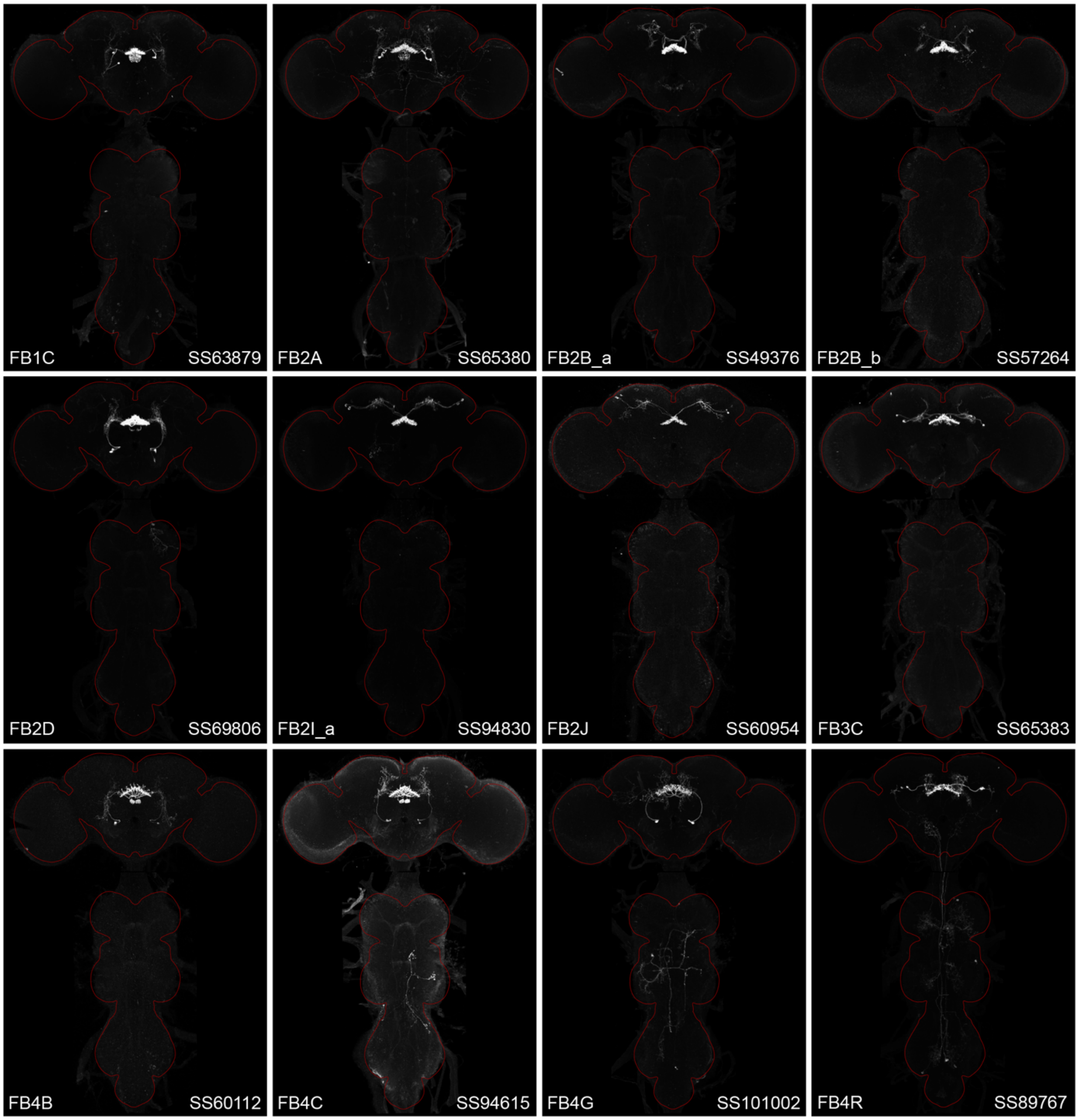


**Figure 2—figure supplement 1**. Maximum intensity projections (20X confocal images) of the expression patterns driven by stable split-GAL4 lines for the indicated twelve cell types. The brain and VNC are outlined in red. Original confocal stacks that include a neuropil reference channel can be downloaded from www.janelia.org/split-gal4.

bioRxiv preprint doi: https://doi.org/10.1101/2024.10.21.619448; this version posted October 22, 2024. The copyright holder for this preprint (which was not certified by peer review) is the author/funder, who has granted bioRxiv a license to display the preprint in perpetuity. It is made available under aCC-BY 4.0 International license.

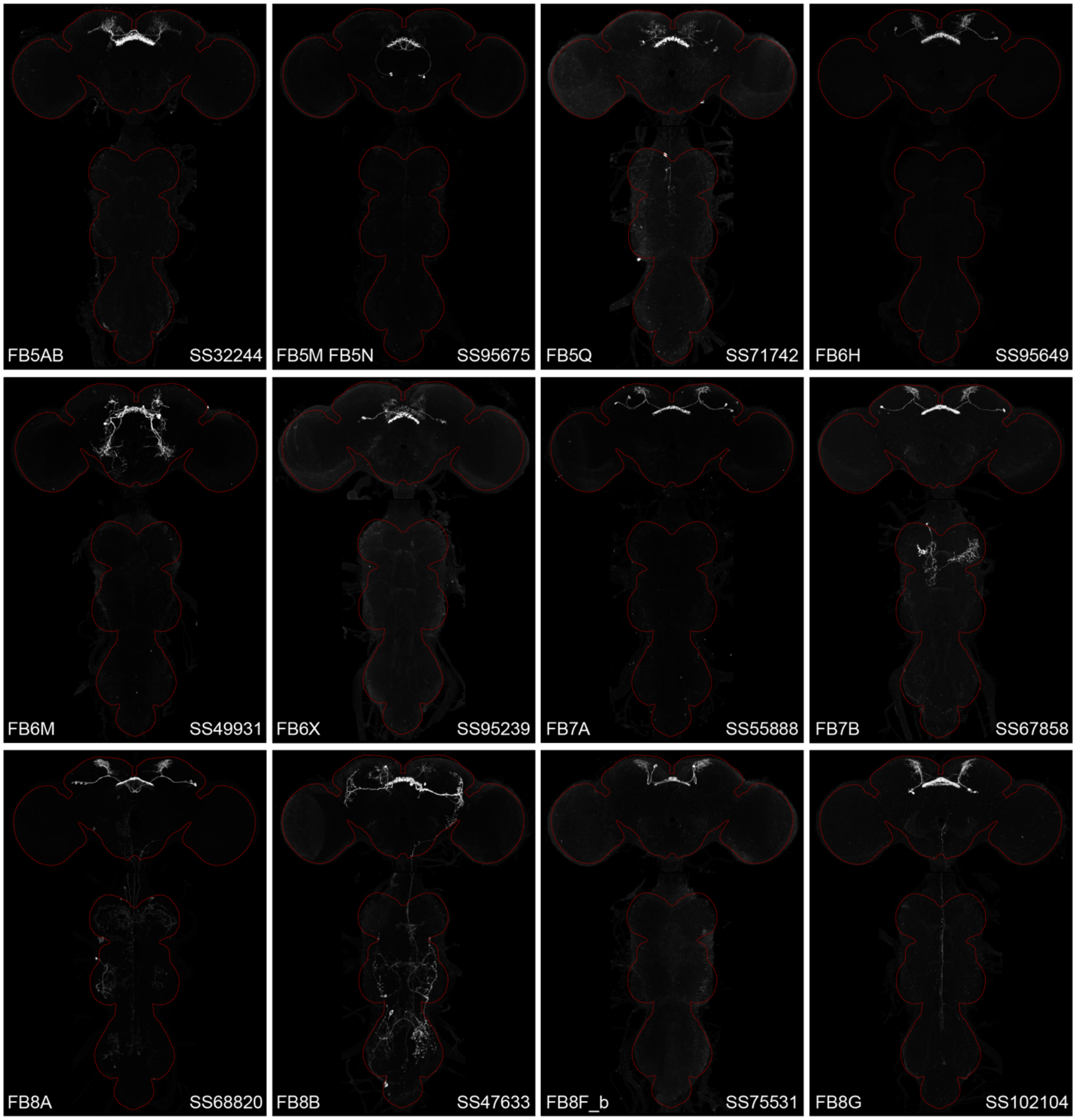


**Figure 2—figure supplement 2**. Maximum intensity projections (20X confocal images) of the expression patterns driven by stable split-GAL4 lines for the indicated twelve cell types. The brain and VNC are outlined in red. Original confocal stacks that include a neuropil reference channel can be downloaded from www.janelia.org/split-gal4.

bioRxiv preprint doi: https://doi.org/10.1101/2024.10.21.619448; this version posted October 22, 2024. The copyright holder for this preprint (which was not certified by peer review) is the author/funder, who has granted bioRxiv a license to display the preprint in perpetuity. It is made available under aCC-BY 4.0 International license.

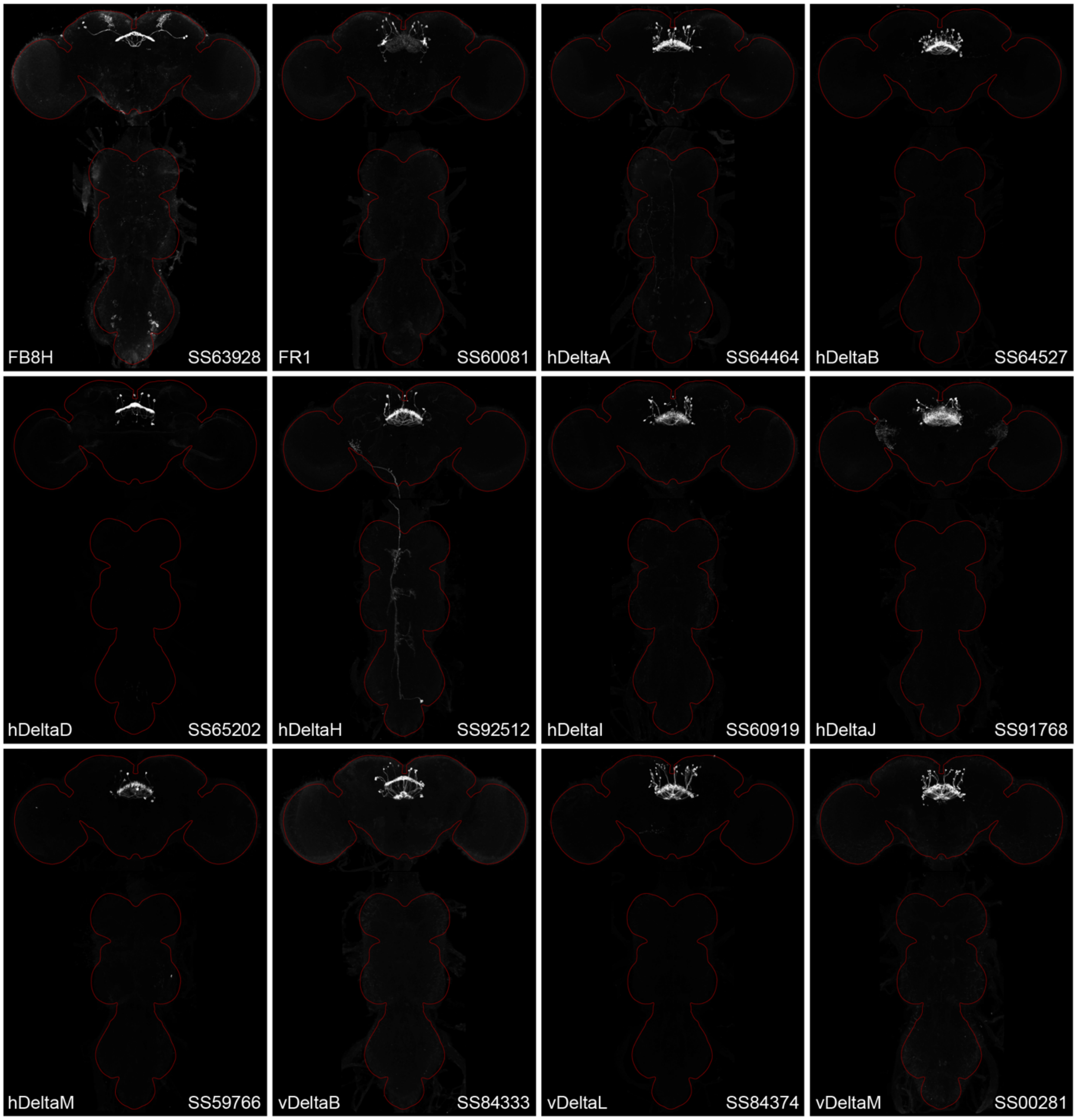


**Figure 2—figure supplement 3**. Maximum intensity projections (20X confocal images) of the expression patterns driven by stable split-GAL4 lines for the indicated twelve cell types. The brain and VNC are outlined in red. Original confocal stacks that include a neuropil reference channel can be downloaded from www.janelia.org/split-gal4.

bioRxiv preprint doi: https://doi.org/10.1101/2024.10.21.619448; this version posted October 22, 2024. The copyright holder for this preprint (which was not certified by peer review) is the author/funder, who has granted bioRxiv a license to display the preprint in perpetuity. It is made available under aCC-BY 4.0 International license.

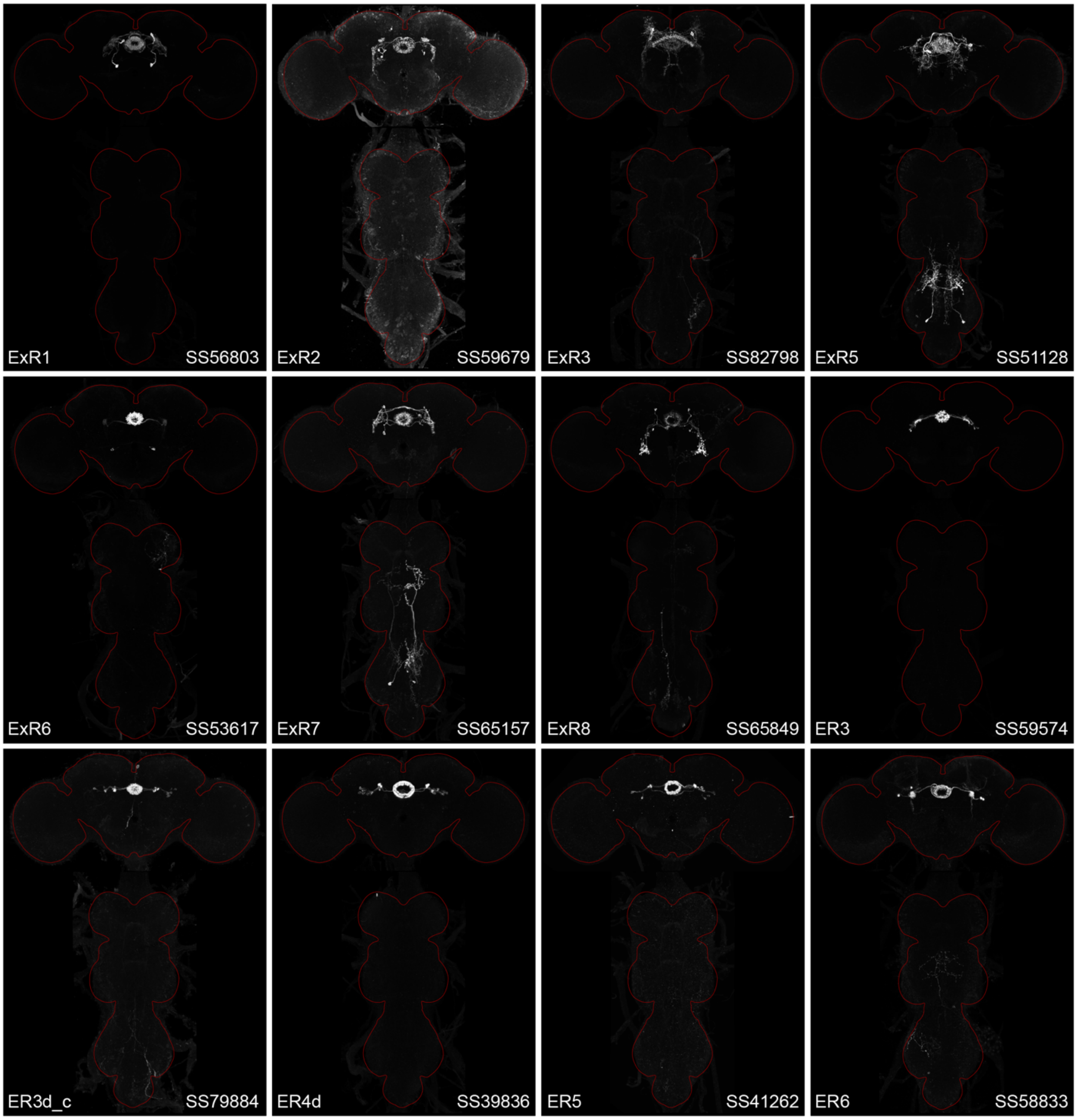


**Figure 2—figure supplement 4.** Maximum intensity projections (20X confocal images) of the expression patterns driven by stable split-GAL4 lines for the indicated twelve cell types. The brain and VNC are outlined in red. Original confocal stacks that include a neuropil reference channel can be downloaded from www.janelia.org/split-gal4.

bioRxiv preprint doi: https://doi.org/10.1101/2024.10.21.619448; this version posted October 22, 2024. The copyright holder for this preprint (which was not certified by peer review) is the author/funder, who has granted bioRxiv a license to display the preprint in perpetuity. It is made available under aCC-BY 4.0 International license.

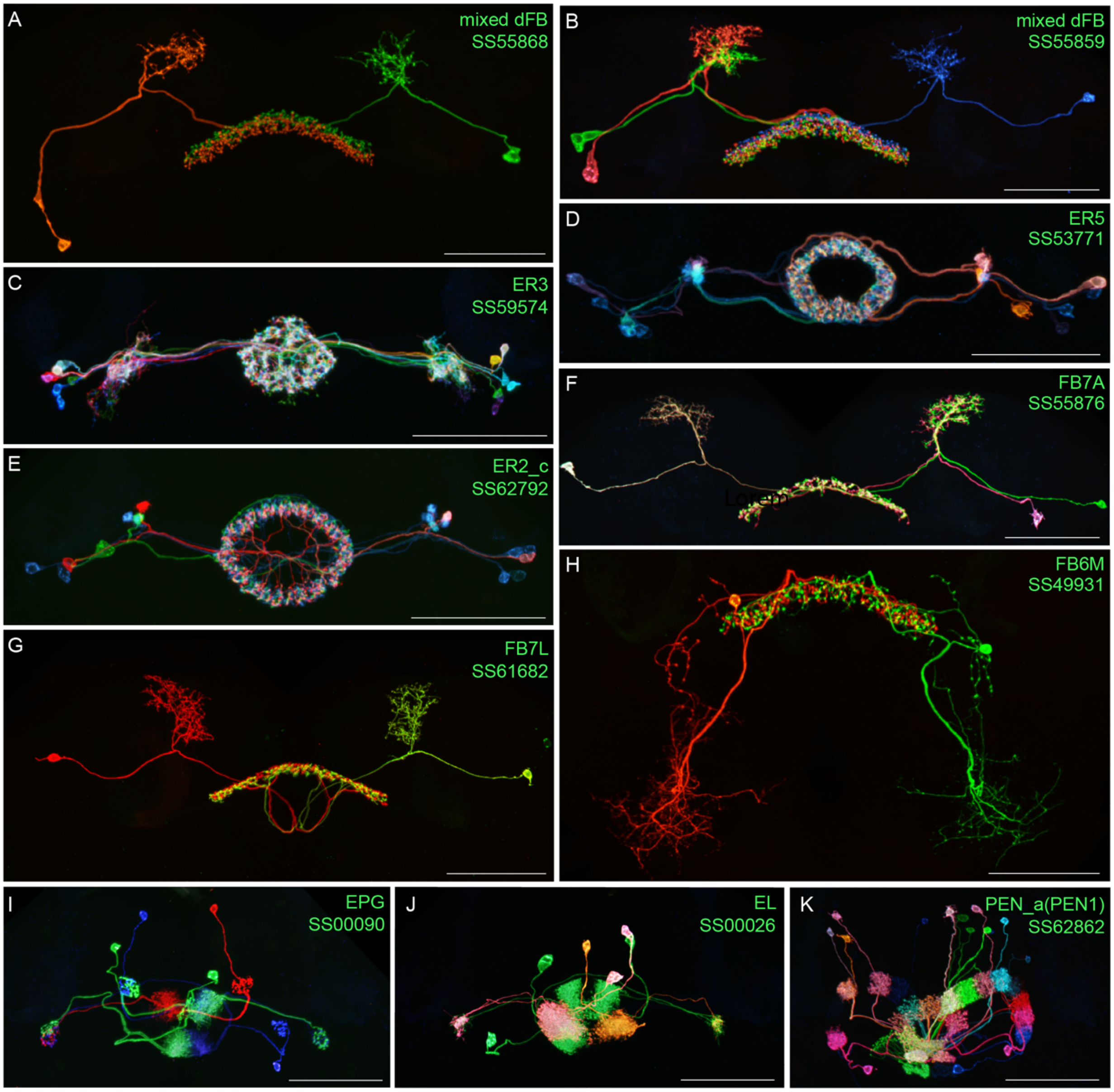


**Figure 3—figure supplement 1**. Individual cells within the indicated cell types revealed by stochastic labeling using the MCFO method (Nern et al. 2015) with the indicated split-GAL4 lines. Images are MIPs. Scale bars = 50 μm.

bioRxiv preprint doi: https://doi.org/10.1101/2024.10.21.619448; this version posted October 22, 2024. The copyright holder for this preprint (which was not certified by peer review) is the author/funder, who has granted bioRxiv a license to display the preprint in perpetuity. It is made available under aCC-BY 4.0 International license.

**Neuropeptides**

| Symbol | Name | Figures | Notes |
|---|---|---|---|
| Akh | Adipokinetic hormone | | negative |
| amn | amnesiac | | negative |
| AstA | Allatostatin A | 6A, 8C | CX candidate |
| AstC | Allatostatin C | 6B, 8D | CX candidate |
| AstCC | Allatostatin double C | | not tested |
| Burs | Bursicon | | not tested |
| Capa | Capability | 5A | |
| CCAP | Crustacean cardioactive peptide | 5B | CX candidate |
| CCHa1 | CCHamide-1 | 6C | CX candidate |
| CCHa2 | CCHamide-2 | 6D | CX candidate |
| CNMa | CNMamide | 5C | |
| Crz | Corazonin | 5D | |
| Dh31 | Diuretic hormone 31 | 6E, 8G, 8N, video 1 | CX candidate |
| Dh44 | Diuretic hormone 44 | 6F, 8P | CX candidate |
| Dsk | Drosulfakinin | 5H, 5I | |
| Eh | Eclosion hormone | 5J | |
| ETH | Ecdysis triggering hormone | | not tested |
| FMRFa | FMRFamide | 6G, 8Q | CX candidate |
| Gpa2 | Glycoprotein hormone alpha 2 | | not tested |
| Gpa5 | Glycoprotein hormone beta 5 | | negative |
| Hug | Hugin | 5K | |
| Ilp1 | Insulin-like peptide 1 | | negative |
| Ilp2 | Insulin-like peptide 2 | 5E | |
| Ilp3 | Insulin-like peptide 3 | 5F | |
| Ilp4 | Insulin-like peptide 4 | | negative |
| Ilp5 | Insulin-like peptide 5 | 5G | |
| Ilp6 | Insulin-like peptide 6 | | negative |
| Ilp7 | Insulin-like peptide 7 | | not tested |
| Ilp8 | Insulin-like peptide 8 | | not tested |
| ITP | Ion transport peptide | 5L | |
| Lk | Leucokinin | 5M | |
| Mip | Myoinhibiting peptide precursor | 6H, 8F, 8H | CX candidate |
| Ms | Myosuppressin | 6I | CX candidate |
| Natalisin | Natalisin | 5N | |
| NPF | neuropeptide F | 6J, 6K, 8A, 8B | CX candidate |
| Nplp1 | Neuropeptide-like precursor 1 | 6L, 8R | CX candidate |
| Nplp2 | Neuropeptide-like precursor 2 | | negative |

**Neuropeptides (cont.)**

| Symbol | Name | Figures | Notes |
|---|---|---|---|
| Nplp3 | Neuropeptide-like precursor 3 | | not tested |
| Nplp4 | Neuropeptide-like precursor 4 | | not tested |
| Orcokinin | Orcokinin | 5O | |
| Pburs | Partner of Bursicon | | not tested |
| Pdf | Pigment-dispersing factor | 5P | |
| Proc | Proctolin | 6M, 8M | CX candidate |
| Ptth | Prothoracicotropic hormone | | not tested |
| RYa | RYamide | | not tested |
| SIFa | SIFamide | 5Q, 8O | CX candidate |
| sNPF | short neuropeptide F precursor | 6N, 8L | CX candidate |
| SP | Sex Peptide | | not tested |
| spab | space blanket | 6O, 8K | CX candidate |
| Tk | Tachykinin | 6P, 8E, 8I, 8J | CX candidate |
| Trissin | Trissin | 5R | |

**Neuropeptide Receptors**

| Symbol | Name | Figures |
|---|---|---|
| AstA-R1 | Allatostatin A receptor 1 | 7A, 7B |
| AstC-R1 | Allatostatin C receptor 1 | 7C |
| CNMaR | CNMamide Receptor | 7D, 7E |
| Dh31-R | Diuretic hormone 31 Receptor | 7F |
| Dh44-R1 | Diuretic hormone 44 receptor 1 | 7G |
| Dh44-R2 | Diuretic hormone 44 receptor 2 | 7G, video 1 |
| FMRFaR | FMRFamide Receptor | 7H |
| hec | hector | 7I |
| Lgr1 | Leucine-rich repeat-containing G protein-coupled receptor 1 | 7J, 7K |
| NPFR | Neuropeptide F receptor | 7L |
| Pdfr | Pigment-dispersing factor receptor | 7M, 7N, video 1 |
| PK1-R | Pyrokinin 1 receptor | 7O |
| ProcR | Proctolin receptor | 7P |
| SifaR | SIFamide receptor | 7Q |
| sNPF-R | short neuropeptide F receptor | 7R |
| SPR | Sex peptide receptor | 7S |
| TKR86C | Tachykinin-like receptor at 86C | 7T |
| TKR99D | Tachykinin-like receptor at 99D | 7T |

**Figure 5—figure supplement 1**. Neuropeptides and neuropeptide receptor genes whose expression patterns were characterized by the EASI-FISH experiments shown in Figures 5-8. Relevant figure panels are listed. Some predicted neuropeptides were not tested because of lack of evidence for their expression in adult brain and are shown in grey font and listed as "not tested" in the Notes column. Neuropeptides shaded in green were tested against split-GAL4 lines with expression in CX cell types (see Figures 8 and 9) based on their cell body positions being in the same brain areas as those of CX cell types. These are listed as "CX candidate" in the Notes column. Those neuropeptides shown in blue font were tested in adult brains, but expression was not detected.; these are listed as "negative" in the Notes column. Lines for which the Notes column is blank were detected in the adult brain but were not chosen for screening against split-GAL4 lines based on the position of their cell bodies.

bioRxiv preprint doi: https://doi.org/10.1101/2024.10.21.619448; this version posted October 22, 2024. The copyright holder for this preprint (which was not certified by peer review) is the author/funder, who has granted bioRxiv a license to display the preprint in perpetuity. It is made available under aCC-BY 4.0 International license.

**Neurotransmitters**

| Gene | Cell Type: ER5 | ExR1 | hDeltaK | FB6A | FB6A | FB7A | FB2 |
|---|---|---|---|---|---|---|---|
| ChAT | 7.51 | 10.05 | 10.81 | 6.95 | 7.83 | 8.20 | 8.70 |
| VAChT | 6.06 | 8.66 | 8.63 | 5.40 | 5.83 | 6.71 | 6.15 |
| VGlut | 5.64 | 3.46 | 5.25 | 8.99 | 8.75 | 9.47 | 9.16 |
| Gad1 | 10.60 | 4.80 | 6.38 | 3.46 | 4.46 | 5.23 | 4.76 |
| VGAT | 7.95 | 4.25 | 5.68 | 3.55 | 3.10 | 4.27 | 4.92 |
| ple | 0.00 | 0.26 | 0.00 | 3.73 | 4.02 | 0.00 | -1.17 |
| Ddc | 9.09 | 7.70 | 10.75 | 7.93 | 6.51 | 8.43 | 9.53 |
| DAT | 2.22 | 4.00 | 0.00 | 8.50 | 7.16 | 7.81 | 3.04 |
| Vmat | 0.00 | 3.66 | 4.71 | 9.81 | 4.73 | 1.81 | 8.05 |
| Trh | 1.87 | 1.77 | 4.02 | 6.24 | 6.72 | 8.55 | 6.32 |
| SerT | 0.00 | -0.74 | 0.00 | -0.42 | -1.32 | -1.00 | 5.07 |
| Tbh | 8.46 | 1.26 | 6.96 | 6.83 | 3.14 | 4.15 | 5.91 |
| Tdc1 | 0.00 | 0.00 | -2.32 | -2.00 | 1.93 | -2.00 | 0.00 |
| Tdc2 | 0.00 | -0.32 | -0.74 | 4.29 | -2.32 | -2.00 | 0.00 |
| prt | 6.08 | 0.49 | 7.79 | 6.10 | 2.26 | 4.36 | 5.68 |

**Neurotransmitter Receptors**

| Gene | Cell Type: ER5 | ExR1 | hDeltaK | FB6A | FB6A | FB7A | FB2 |
|---|---|---|---|---|---|---|---|
| mAChR-A | 9.42 | 7.91 | 7.79 | 5.29 | 5.69 | 7.95 | 8.08 |
| mAChR-B | 3.32 | 1.49 | 0.00 | 3.52 | 4.07 | 7.54 | 2.42 |
| mAChR-C | 0.00 | 0.00 | 0.00 | 0.00 | 0.00 | 0.00 | 0.00 |
| nAChRalpha1 | 6.87 | 6.54 | 6.77 | 4.74 | 4.05 | 5.24 | 5.11 |
| nAChRalpha2 | 5.38 | 5.97 | 4.60 | 0.58 | 5.15 | 5.63 | 5.53 |
| nAChRalpha3 | 6.14 | 9.06 | 6.08 | 6.88 | 6.04 | 6.25 | 7.66 |
| nAChRalpha4 | 6.31 | 6.00 | 7.25 | 4.43 | 4.51 | 5.06 | 5.42 |
| nAChRalpha5 | 9.70 | 7.54 | 9.07 | 7.92 | 6.66 | 8.31 | 8.34 |
| nAChRalpha6 | 12.55 | 7.75 | 10.99 | 9.37 | 9.70 | 10.13 | 9.84 |
| nAChRalpha7 | 11.94 | 7.71 | 9.04 | 7.69 | 6.20 | 6.79 | 7.31 |
| nAChRbeta1 | 9.77 | 10.50 | 9.66 | 8.55 | 8.46 | 9.04 | 10.33 |
| nAChRbeta2 | 7.74 | 6.41 | 7.10 | 5.55 | 5.68 | 6.44 | 7.14 |
| nAChRbeta3 | 0.00 | 0.00 | 0.00 | -2.00 | -2.32 | 0.00 | 0.00 |
| GABA-B-R1 | 4.32 | 7.77 | 7.44 | 6.41 | 6.31 | 6.32 | 6.71 |
| GABA-B-R2 | 6.60 | 6.50 | 7.05 | 5.52 | 4.41 | 7.20 | 6.27 |
| GABA-B-R3 | 4.00 | 3.83 | 3.51 | 1.00 | 0.26 | 4.44 | 5.68 |
| Rdl | 12.88 | 12.14 | 11.15 | 10.27 | 10.28 | 10.82 | 11.82 |
| Lcch3 | 6.38 | 6.08 | 6.12 | 5.54 | 4.12 | 3.93 | 6.07 |

-3.0 9.0 14

**Neurotransmitter Receptors**

| Gene | Cell Type: ER5 | ExR1 | hDeltaK | FB6A | FB6A | FB7A | FB2 |
|---|---|---|---|---|---|---|---|
| GluRIA | 2.94 | 5.53 | 6.51 | 8.29 | 8.04 | 6.87 | 4.81 |
| GluRIIA | -1.58 | 0.85 | 0.00 | 1.17 | 0.00 | 0.00 | 0.00 |
| GluRIB | 4.68 | 7.58 | 6.04 | 9.15 | 8.70 | 7.40 | 4.66 |
| GluRIIB | 0.00 | 0.00 | -2.32 | 0.00 | 0.00 | -2.00 | -0.85 |
| GluRIIC | 0.00 | -2.32 | -2.32 | 0.00 | 1.26 | 0.00 | -1.58 |
| GluRIID | 0.00 | 0.00 | 0.00 | -2.00 | 2.63 | 0.00 | 0.00 |
| GluRIIE | 0.00 | 0.00 | -2.32 | -2.00 | -0.74 | 0.00 | 0.00 |
| mGluR | 5.68 | 1.00 | 4.38 | 5.04 | 1.49 | 4.19 | 4.98 |
| dmGlut | 0.00 | 0.00 | 0.00 | 1.32 | -0.74 | 0.00 | -2.17 |
| GluClalpha | 6.07 | 10.14 | 7.88 | 7.09 | 7.01 | 7.50 | 7.98 |
| CG11155 | 8.64 | 7.48 | 7.93 | 6.82 | 6.78 | 8.16 | 8.13 |
| KaiR1D | 6.65 | 4.66 | 5.78 | 4.70 | 3.81 | 6.29 | 6.07 |
| Ekar | 0.00 | -0.74 | 1.77 | 0.32 | 1.38 | 3.46 | -0.36 |
| clumsy | 0.00 | 2.38 | 0.00 | -2.00 | -0.32 | 0.00 | -3.17 |
| Grik | 4.20 | 3.23 | 4.02 | 3.49 | -1.32 | 3.32 | -0.85 |
| Nmdar1 | 3.81 | 4.64 | 1.00 | 3.75 | 2.93 | 6.30 | 5.46 |
| Nmdar2 | 7.68 | 7.49 | 6.14 | 6.13 | 6.01 | 5.68 | 5.45 |
| GlyT | 5.39 | 6.92 | 5.93 | 5.33 | 4.15 | 3.64 | 6.43 |
| Grd | 0.00 | -2.32 | 1.68 | 2.81 | 5.28 | 6.67 | 2.22 |
| SecCl | 0.00 | 4.41 | 0.00 | -0.42 | -2.32 | 0.00 | 0.00 |
| CG12344 | 0.00 | 0.00 | 0.00 | 0.58 | -0.32 | 3.73 | -2.17 |
| Hdc | 0.00 | -0.74 | 0.00 | 0.00 | -0.32 | 0.00 | -2.17 |
| ort | -1.58 | -0.74 | 0.00 | 1.46 | 1.68 | 0.00 | 2.04 |
| HisCl1 | 0.00 | -0.74 | 0.00 | -2.00 | -0.32 | 0.00 | -3.17 |
| Dop1R1 | 3.97 | 6.38 | 6.87 | 8.39 | 9.04 | 7.74 | 6.64 |
| Dop1R2 | 3.84 | 5.60 | 2.43 | 4.43 | 5.05 | 5.61 | 7.29 |
| Dop2R | 6.74 | 8.48 | 9.32 | 9.63 | 7.86 | 9.53 | 9.15 |
| DopEcR | 8.04 | 8.04 | 6.95 | 7.33 | 6.58 | 5.78 | 7.26 |
| Oamb | 9.92 | 3.87 | 5.66 | 3.17 | 3.68 | 3.88 | 4.12 |
| Octbeta1R | 4.72 | 6.84 | 5.42 | 9.85 | 9.20 | 10.46 | 4.77 |
| Octbeta2R | 2.58 | 5.09 | 7.46 | 3.64 | 4.72 | 3.29 | 3.61 |
| Octbeta3R | 3.77 | 5.50 | 5.19 | 6.40 | 6.89 | 7.71 | 4.70 |
| Oct-TyrR | 5.47 | 6.33 | 0.85 | 1.91 | 0.26 | 0.58 | 5.79 |
| TyrR | 1.00 | 1.14 | 0.49 | 3.09 | 1.26 | 0.58 | 1.69 |
| TyrRII | 0.00 | -2.32 | 0.00 | 0.32 | -2.32 | 1.70 | 0.00 |
| 5-HT1A | 6.70 | 5.94 | 5.15 | 5.14 | 2.32 | 4.44 | 7.41 |
| 5-HT1B | -1.58 | 4.78 | 1.49 | 2.58 | 4.70 | -2.00 | 4.93 |
| 5-HT2A | 8.48 | 2.93 | 4.34 | 7.92 | 7.11 | 7.26 | 3.98 |
| 5-HT2B | 0.00 | 4.43 | 8.22 | 6.78 | 5.60 | -0.42 | 3.02 |
| 5-HT7 | 6.00 | 3.17 | 5.49 | 5.99 | 4.70 | 4.51 | 0.74 |

**Figure 9—figure supplement 1.** RNA-seq data for genes related to neurotransmitter synthesis, transport, and receptors in the indicated cell types. Mean $log_2$ of TPM values for each gene are listed. See NCBI Gene Expression Omnibus (accession number GSE271123) for the raw data. The split-GAL4 drivers used for each cell type were as follows: ER5, SS00070; ExR1, SS56684; hDeltaK, SS02748; FB6A, SS57656 and SS54343; FB7A, SS55888; FB2, SS56319.

bioRxiv preprint doi: https://doi.org/10.1101/2024.10.21.619448; this version posted October 22, 2024. The copyright holder for this preprint (which was not certified by peer review) is the author/funder, who has granted bioRxiv a license to display the preprint in perpetuity. It is made available under aCC-BY 4.0 International license.

Neuropeptides

| Gene | Cell Type | | | | | | |
|---|---|---|---|---|---|---|---|
| | ER5 | ExR1 | hDeltaK | FB6A | FB6A | FB7A | FB2 |
| Akh | 0.00 | 0.85 | 0.00 | -2.00 | 2.07 | 2.25 | 2.26 |
| amn | 1.58 | -1.32 | 4.14 | 0.00 | 0.00 | -0.42 | 1.08 |
| AstA | 1.22 | 0.49 | 13.49 | 1.32 | -2.32 | 0.00 | -3.17 |
| AstC | 5.55 | 0.49 | 6.94 | 8.51 | 12.28 | 5.02 | -1.58 |
| Burs | 0.00 | 0.00 | 0.00 | 0.00 | 0.00 | 0.00 | 0.00 |
| Capa | 0.00 | -0.74 | -0.32 | 0.00 | 0.00 | 0.00 | 0.00 |
| CCAP | 0.00 | 0.00 | 0.00 | 0.00 | -0.74 | -1.00 | 0.00 |
| CCHa1 | 0.00 | -0.32 | 0.00 | 0.00 | -2.32 | 0.00 | -1.58 |
| CCHa2 | 0.00 | 0.68 | 1.58 | 2.70 | -0.74 | 0.00 | 2.32 |
| CNMa | 0.00 | -1.32 | 0.00 | -2.00 | 0.00 | -2.00 | 0.00 |
| Crz | 0.00 | 0.00 | -0.74 | 0.00 | -2.32 | 0.00 | 6.63 |
| Dh31 | 12.77 | 3.77 | 7.85 | 0.00 | 3.94 | 0.00 | 4.81 |
| Dh44 | 2.74 | 4.70 | 5.98 | 5.33 | 1.26 | 0.00 | 1.64 |
| Dsk | 0.00 | 0.00 | 0.00 | 0.00 | -1.32 | 0.00 | 0.00 |
| Eh | 0.00 | 0.00 | 0.00 | -2.00 | 2.07 | 0.00 | 0.00 |
| ETH | 0.00 | 0.00 | 0.00 | 0.00 | 2.58 | 0.00 | 1.87 |
| FMRFa | 0.00 | -0.74 | -2.32 | 0.00 | 0.00 | -2.00 | 0.92 |
| Hug | 0.00 | 0.00 | 0.00 | 0.00 | 0.26 | 0.00 | 1.35 |
| ITP | 0.00 | 4.32 | 3.41 | 2.09 | 0.26 | 0.00 | -0.17 |
| Lk | 0.00 | -2.32 | 0.00 | 0.00 | 0.00 | 0.00 | 0.00 |
| Mip | 5.32 | 1.58 | 0.00 | 5.39 | 5.37 | 5.86 | 12.94 |
| Ms | 0.00 | 3.35 | 0.00 | 3.93 | 1.00 | -1.00 | 3.15 |
| NPF | 0.00 | -2.32 | 12.02 | 0.00 | -2.32 | 0.00 | -3.17 |
| Nplp1 | 8.05 | 4.07 | 4.57 | 7.68 | 5.86 | 9.17 | 8.34 |
| Nplp3 | 0.00 | 0.00 | -2.32 | 0.00 | 0.00 | 0.00 | 0.00 |
| Nplp4 | 0.00 | 0.00 | 0.00 | 0.00 | 1.38 | 0.00 | 0.00 |
| Orcokinin | 0.00 | 0.00 | 0.00 | 0.00 | -1.32 | 3.98 | 3.58 |
| Pburs | 0.00 | 0.00 | 0.00 | 0.00 | -2.32 | 0.00 | 0.00 |
| Pdf | 0.00 | -2.32 | 0.00 | 0.00 | -0.74 | -2.00 | 0.42 |
| Proc | 4.39 | 4.63 | 8.69 | 3.17 | -0.32 | 0.81 | 2.12 |
| Ptth | 0.00 | 0.00 | 0.00 | 0.00 | 0.00 | 0.00 | -3.17 |
| RYa | 0.74 | 0.00 | -1.32 | 6.46 | 6.06 | 9.07 | 5.90 |
| SIFa | 0.00 | 0.00 | 0.00 | 0.00 | -2.32 | 0.00 | 0.00 |
| sNPF | 5.40 | 5.31 | 6.64 | 4.02 | 3.72 | 3.39 | 3.27 |
| Tk | 0.00 | 3.93 | 4.22 | 4.92 | 2.89 | 5.13 | 13.63 |
| Trissin | 0.00 | 1.68 | -2.32 | 0.00 | -1.32 | 0.00 | 1.08 |

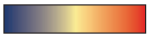


Neuropeptide Receptors

| Gene | Cell Type | | | | | | |
|---|---|---|---|---|---|---|---|
| | ER5 | ExR1 | hDeltaK | FB6A | FB6A | FB7A | FB2 |
| AkhR | 0.00 | -1.32 | 3.41 | 0.00 | -1.32 | 0.00 | -1.17 |
| AstA-R1 | -1.58 | 2.07 | 9.23 | 6.60 | 4.95 | 8.06 | 4.31 |
| AstA-R2 | 0.00 | 4.89 | 0.00 | 0.58 | 4.62 | 0.00 | 0.00 |
| AstC-R1 | 5.00 | 0.68 | 0.85 | 2.46 | -0.74 | -1.00 | 2.71 |
| AstC-R2 | 4.27 | 6.50 | 1.14 | 1.70 | 2.58 | -0.42 | 0.00 |
| CapaR | 0.00 | 0.00 | 0.00 | -0.42 | -2.32 | 0.00 | -1.58 |
| CCAP-R | 0.00 | -0.32 | -2.32 | 0.00 | -0.32 | 0.00 | 5.85 |
| CCHa1-R | 0.00 | 1.68 | 1.38 | 0.00 | 0.00 | 0.00 | 4.60 |
| CCHa2-R | 0.00 | -1.32 | -1.32 | 0.00 | 0.00 | -2.00 | 2.50 |
| CCKLR-17D1 | 0.00 | -0.74 | 3.96 | 1.17 | -2.32 | 0.00 | -0.36 |
| CCKLR-17D3 | 0.00 | 1.58 | 4.52 | 2.70 | 2.54 | 3.04 | 1.15 |
| CG10738 | -1.58 | -0.32 | 1.49 | 0.58 | 3.29 | 4.81 | 4.10 |
| CG13229 | 0.00 | 3.23 | 4.47 | -0.42 | 4.36 | 2.81 | 3.27 |
| CG13575 | 0.00 | 0.00 | -2.32 | 0.00 | 0.00 | 0.00 | 0.00 |
| CG13579 | 0.00 | 0.00 | 0.00 | 2.70 | -0.32 | 5.99 | 2.96 |
| CG13995 | 7.67 | 6.51 | 7.86 | 4.95 | 5.28 | 5.70 | 6.45 |
| CG30340 | -1.58 | -0.32 | 0.00 | 1.81 | -2.32 | -2.00 | -3.17 |
| CG32547 | 6.55 | 5.97 | 5.26 | 3.55 | -1.32 | -2.00 | 0.92 |
| CG33639 | 2.94 | 5.41 | 7.85 | 4.48 | 3.94 | 3.75 | 6.30 |
| CNMaR | -1.58 | 6.26 | 7.94 | 5.61 | 3.41 | 3.75 | 10.69 |
| CrzR | 2.00 | 2.54 | 0.00 | 0.32 | -1.32 | 3.88 | -1.17 |
| Dh31-R | 6.31 | 3.66 | 3.54 | 0.00 | -0.32 | 0.00 | 4.88 |
| Dh44-R1 | 3.58 | 7.10 | 2.32 | 3.61 | 1.85 | 4.91 | 4.12 |
| Dh44-R2 | 7.16 | 5.29 | 5.39 | 2.09 | 3.29 | 4.11 | 2.98 |
| ETHR | 6.04 | 4.29 | 3.61 | 4.04 | 5.07 | 2.91 | 4.91 |
| FMRFaR | 0.00 | 6.45 | 3.43 | 3.88 | 2.49 | 0.32 | -0.58 |
| hec | 0.00 | 1.14 | 1.00 | 6.07 | 5.41 | 8.01 | 7.28 |
| Lgr1 | 0.00 | 1.58 | 0.49 | 0.00 | 0.00 | 5.48 | 7.07 |
| Lgr3 | 3.00 | 0.49 | 0.00 | -0.42 | -0.74 | 3.39 | 1.69 |
| Lgr4 | 0.00 | 0.68 | 1.85 | 1.58 | -0.32 | 2.00 | -0.85 |
| Lkr | 0.00 | -2.32 | 0.00 | -1.00 | -0.32 | 0.00 | 0.42 |
| MsR1 | 0.00 | 3.20 | 3.87 | -0.42 | -0.32 | 5.12 | 3.53 |
| MsR2 | 0.00 | 1.68 | 2.93 | 1.81 | -2.32 | 3.21 | -0.85 |
| NPFR | 0.00 | 1.49 | 6.69 | 0.00 | 2.38 | 6.09 | 5.15 |
| Pdfr | 9.92 | 5.72 | 4.00 | 1.70 | 3.51 | 0.58 | 7.27 |
| PK1-R | 0.00 | 0.26 | 0.00 | -2.00 | -1.32 | 0.00 | 0.00 |
| PK2-R1 | 0.00 | 5.51 | -2.32 | 0.00 | -0.74 | 0.00 | 1.47 |
| PK2-R2 | 2.66 | 4.60 | 1.38 | -2.00 | -1.32 | 0.00 | 2.47 |
| Proc-R | 1.22 | 0.00 | 3.20 | 0.00 | -2.32 | 0.00 | 2.58 |
| rk | -1.58 | 3.00 | 3.74 | -1.00 | -0.32 | -1.00 | 3.13 |
| RYa-R | 3.06 | 0.00 | 0.00 | 1.70 | -1.32 | 3.00 | -0.58 |
| SIFaR | 0.00 | 6.07 | 6.00 | 6.63 | 3.72 | -1.00 | 4.97 |
| sNPF-R | 5.00 | 6.42 | 0.00 | 2.00 | -0.74 | 0.00 | 4.03 |
| TkR86C | 4.32 | 0.49 | 0.00 | 1.70 | 0.00 | 0.00 | 4.43 |
| TkR99D | 6.75 | 0.00 | 0.00 | -2.00 | 0.00 | 4.13 | 6.35 |
| TrissinR | 2.00 | 4.50 | 2.32 | 1.00 | 2.89 | 0.00 | 2.53 |

**Figure 9—figure supplement 2.** RNA-seq data for genes related to neuropeptides and their receptors in the indicated cell types.

bioRxiv preprint doi: https://doi.org/10.1101/2024.10.21.619448; this version posted October 22, 2024. The copyright holder for this preprint (which was not certified by peer review) is the author/funder, who has granted bioRxiv a license to display the preprint in perpetuity. It is made available under aCC-BY 4.0 International license.

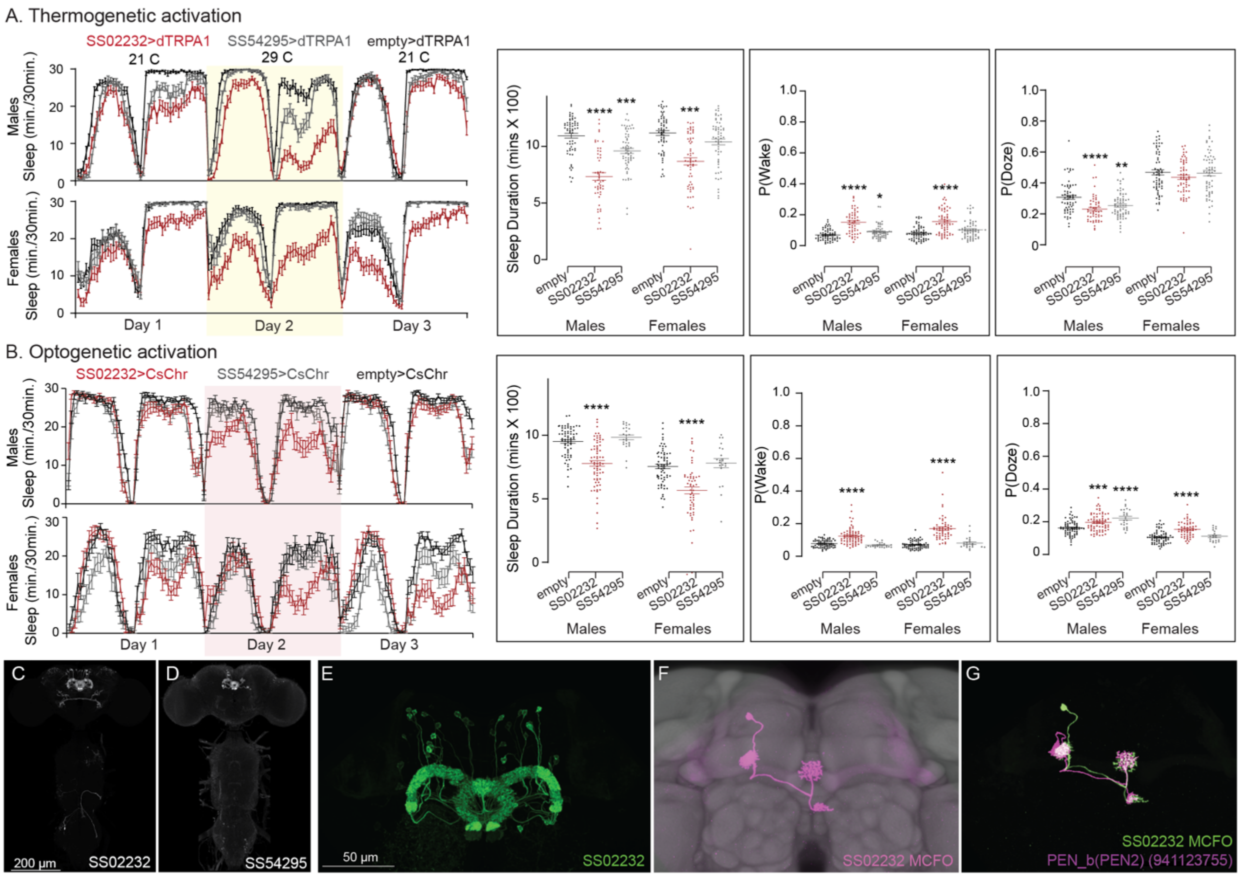


**Figure 10—figure supplement 1**. Activation phenotype of PEN_b, comprised of 22 columnar neurons linking the EB, PB and noduli (Wolff and Rubin 2018; Turner-Evans et al., 2020; Green et al., 2017; Hulse et al., 2021). Effects on activity of thermogenetic (A) and optogenetic (B) activation of PEN_b using split-GAL4 lines SS02232 and SS54295 is shown. Specifically, thermogenetic activation of these split-GAL4 lines decreases sleep duration and increases P(Wake) in males but only SS02232 is wake promoting in females. Optogenetic activation of SS02232 suppresses sleep and increases P(Wake) in both males and females supporting the general function of these neurons as wake-promoting. While both lines show high cell type specificity and minimal VNC expression, SS54295 has stochastic expression that might explain the weaker wake promoting effect seen with this line. (C-D) MIP images of GFP-driven expression in the brain and VNC of the two spilt-GAL4 lines. The brain and VNC are outlined in red. (E) Higher resolution images of the relevant brain area of SS02232. (F) Morphology of a single neuron revealed by stochastic labelling shown with neuropil reference. (G) Comparison of LM and EM cell morphologies. Original confocal stacks for panels C-F can be downloaded from www.janelia.org/split-gal4. The full genotypes of the driver lines are given there and in Supplementary File 1. Statistical comparisons were made by Kruskal-Wallis and Dunn's post hoc test. Asterisk indicates significance from 0: *p<0.05; **p<0.01; ***p<0.001; ****p<0.0001.

bioRxiv preprint doi: https://doi.org/10.1101/2024.10.21.619448; this version posted October 22, 2024. The copyright holder for this preprint (which was not certified by peer review) is the author/funder, who has granted bioRxiv a license to display the preprint in perpetuity. It is made available under aCC-BY 4.0 International license.

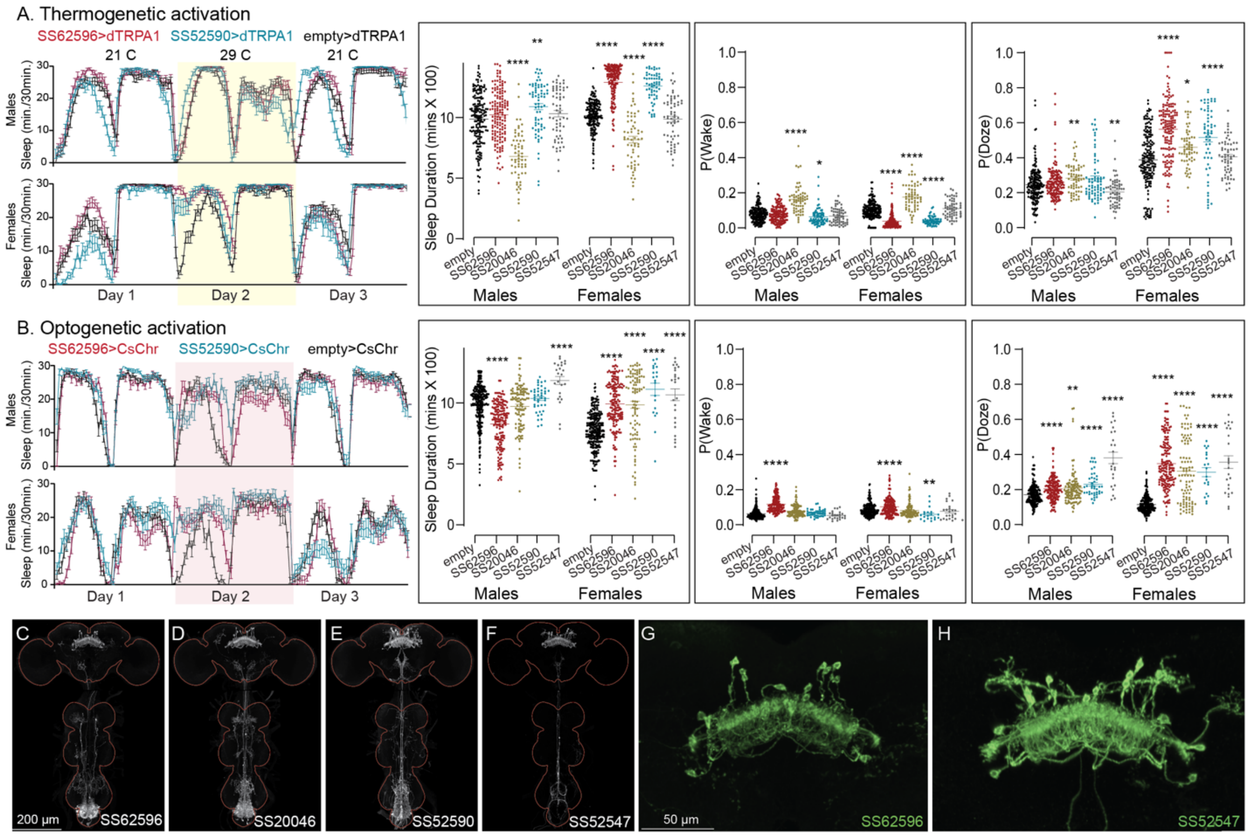


**Figure 10—figure supplement 2.** Activation phenotype of PFGs. Thermogenetic (A) and optogenetic (B) activation of PFGs reveals sexually dimorphic sleep behavior. We tested four different split-GAL4 lines for this cell type. All four lines display expression in the VNC complicating interpretation of the results. To mitigate this issue, we used lines with different hemidriver parents and visually distinct VNC expression patterns (C-F). The brain and VNC are outlined in red. Optogenetic activation (Day 2) in female flies showed increased sleep in all four driver lines and a mild increase in sleep duration in one of lines for male flies (SS52547). However, P(Doze) was consistently higher in male and female flies for all tested PFGs lines. Thermogenetic activation (Day 2) also shows sex specific phenotypes and two of the four lines (SS62596 and SS52590) have increased sleep. SS20046 has decreased sleep in females and male flies with dTRPA1 based activation but shows increased sleep with CsChrimson in females. Despite these inconsistencies, the data suggest a sex specific sleep promoting effect for this cell type. (G-H) Higher resolution images of the relevant brain area of SS62596 and SS52547. Original confocal stacks for panels C-H can be downloaded from www.janelia.org/split-gal4. The full genotypes of the driver lines are given there and in Supplementary File 1. Statistical comparisons were made by Kruskal-Wallis and Dunn's post hoc test. Asterisk indicates significance from 0: *p<0.05; **p<0.01; ***p<0.001; ****p<0.0001.

bioRxiv preprint doi: https://doi.org/10.1101/2024.10.21.619448; this version posted October 22, 2024. The copyright holder for this preprint (which was not certified by peer review) is the author/funder, who has granted bioRxiv a license to display the preprint in perpetuity. It is made available under aCC-BY 4.0 International license.

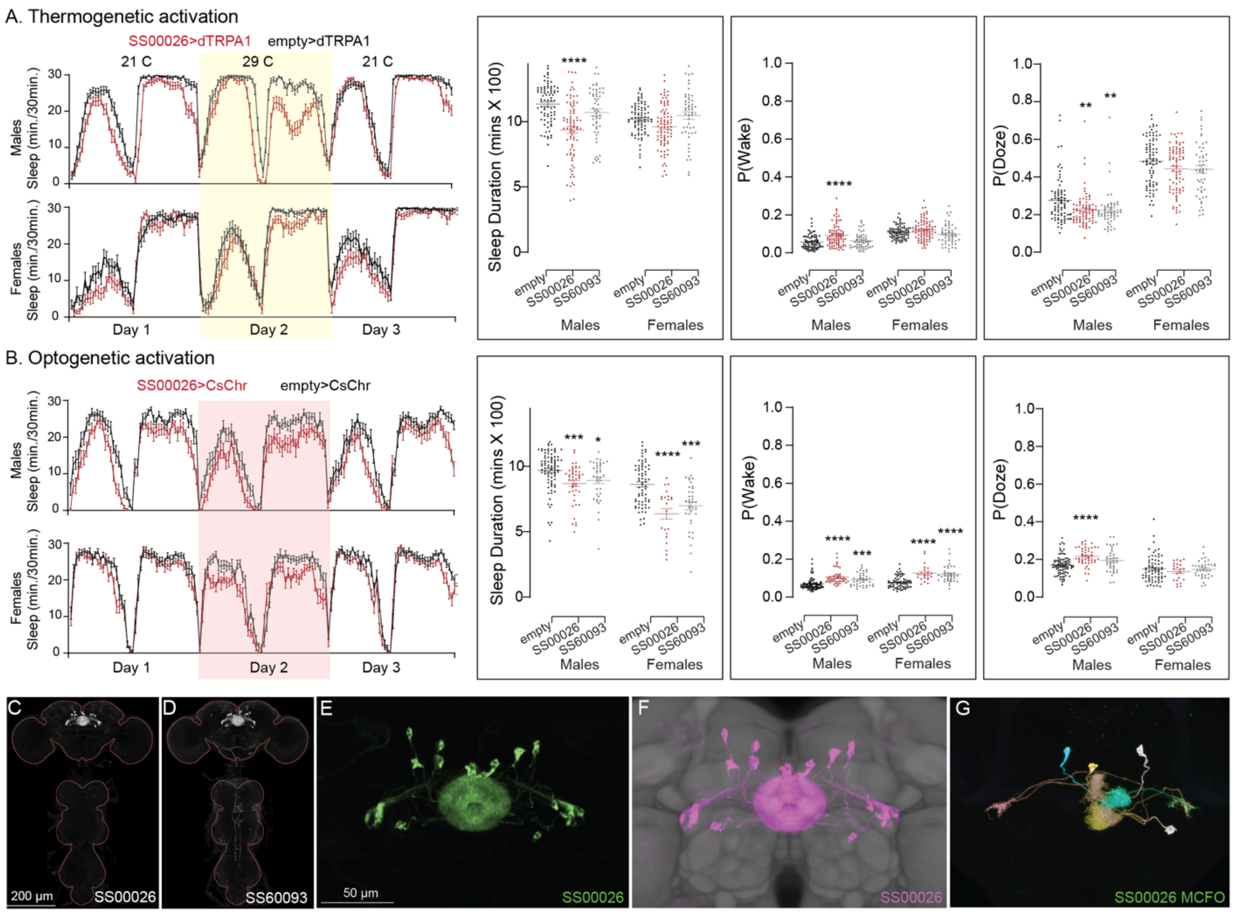


**Figure 10—figure supplement 3**. Thermogenetic activation (A) of EL neurons decreases sleep in one of the split-GAL4 lines (SS00026), while optogenetic activation (B) of both lines decreases sleep and increases P(Wake) on Day 2. (C,D) MIP images of GFP-driven expression in the brain and VNC of the three spilt-GAL4 lines. The brain and VNC are outlined in red. (E) Higher resolution images of the relevant brain area of in SS00026. (F) Same as panel E, but with neuropil reference. (G) Morphology of individual neurons revealed by stochastic labelling. Original confocal stacks for panels C-G can be downloaded from www.janelia.org/split-gal4.. The full genotypes of the driver lines are given there and in Supplementary File 1. Statistical comparisons were made by Kruskal-Wallis and Dunn's post hoc test. Asterisk indicates significance from 0: *p<0.05; **p<0.01; ***p<0.001; ****p<0.0001.

bioRxiv preprint doi: https://doi.org/10.1101/2024.10.21.619448; this version posted October 22, 2024. The copyright holder for this preprint (which was not certified by peer review) is the author/funder, who has granted bioRxiv a license to display the preprint in perpetuity. It is made available under aCC-BY 4.0 International license.

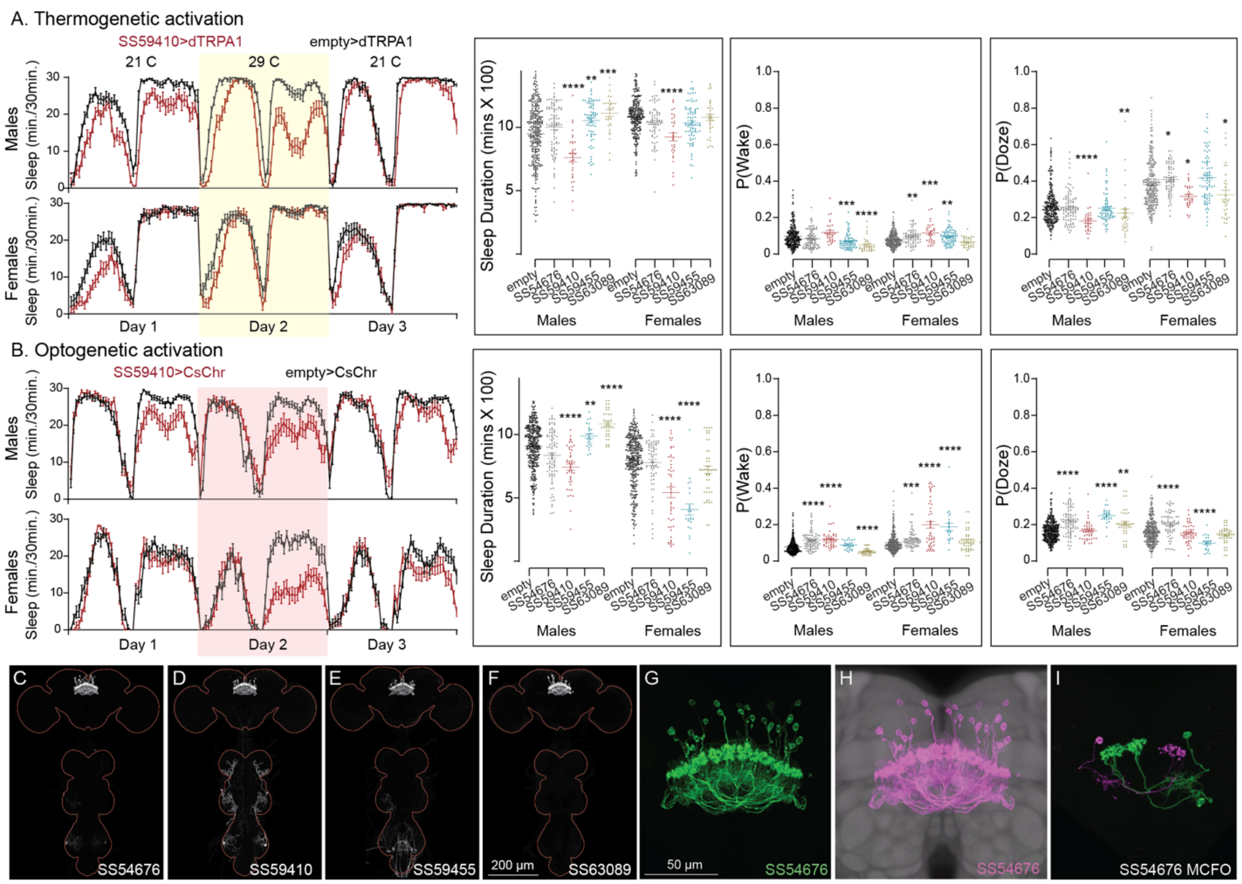


**Figure 10—figure supplement 4**. Activation phenotype of hDeltaK. This group of intrinsic columnar neurons is highly peptidergic and has been implicated in chronic social isolation-induced sleep loss (Li et al., 2021). We tested four split-GAL4 lines (SS59455, SS63089, SS54676 and SS59410). Our results do not give a consistent view of the role of this cell type in sleep regulation. (A) Thermogenetic activation of one of the four lines (SS59410) was wake-promoting in both sexes, while two (SS59455 and SS63089) were sleep-promoting in males, but not females. (B) Consistent to what was observed with thermogenetic activation, optogenetic activation of SS59410 also suppressed sleep in males and females, while SS59410 and SS59455 were sleep promoting in males, but not in females. (C-F) MIP images of GFP-driven expression in the brain and VNC of the four spilt-GAL4 lines. The brain and VNC are outlined in red. (G) Higher resolution images of the relevant brain area of in SS54676 (H) Same as panel G, but with neuropil reference. (I) Morphology of individual neurons revealed by stochastic labelling. We do not have any insight into the differences in behaviors between lines and cannot make an overall conclusion of their role in sleep regulation. Such line-to-line inconsistencies argue for caution in the interpretation of results, especially when only one line for a cell type has been assayed. Original confocal stacks for panels C-I can be downloaded from www.janelia.org/split-gal4. The full genotypes of the driver lines are given there and in Supplementary File 1. Statistical comparisons were made by Kruskal-Wallis and Dunn's post hoc test. Asterisk indicates significance from 0: *p<0.05; **p<0.01; ***p<0.001; ****p<0.0001.

bioRxiv preprint doi: https://doi.org/10.1101/2024.10.21.619448; this version posted October 22, 2024. The copyright holder for this preprint (which was not certified by peer review) is the author/funder, who has granted bioRxiv a license to display the preprint in perpetuity. It is made available under aCC-BY 4.0 International license.

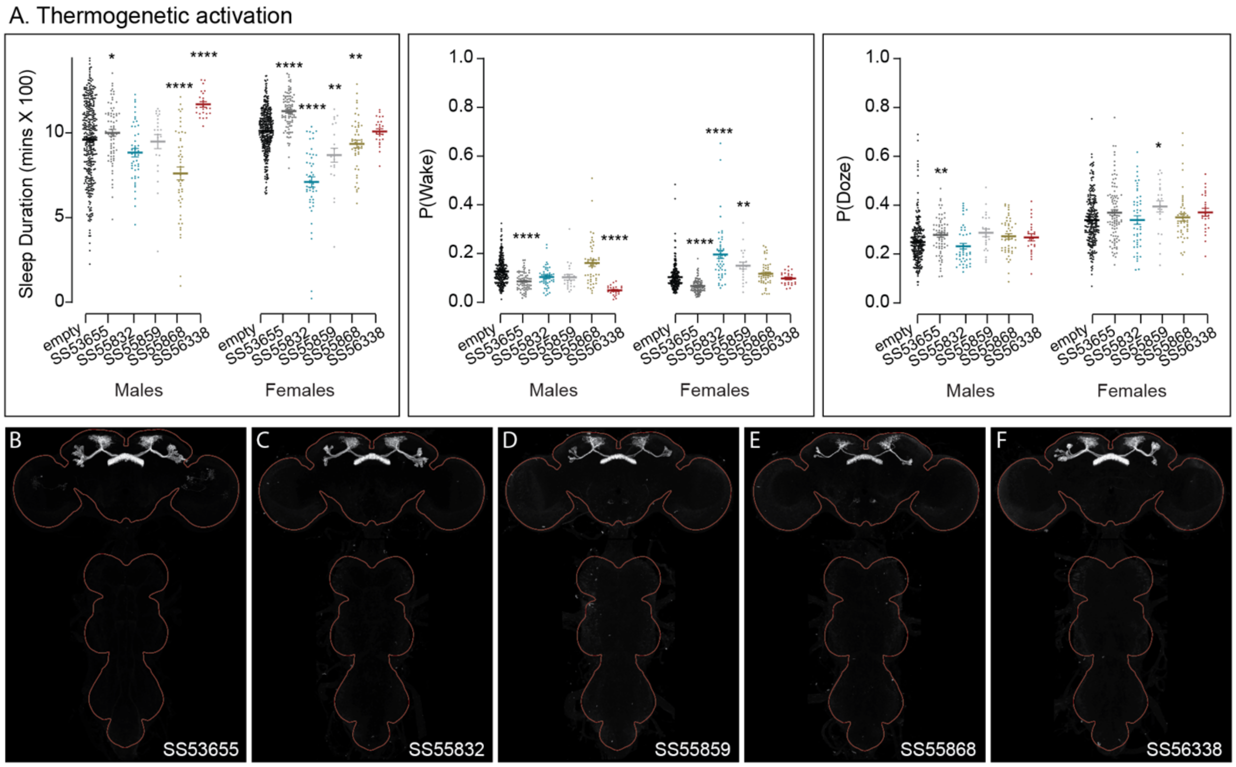


**Figure 10—figure supplement 5**. Activation phenotypes of combinations of dFB cell types. The five split-GAL4 lines used here each utilize R84C10 as one of their hemidrivers. They represent different (but largely overlapping subsets of layer 6 and 7 FB tangential cell types and have no detectable VNC expression. Jones et al. (2024) reported analysis of additional lines that used R84C10 as a hemidriver in split-GAL4 lines. We did not examine expression in other parts of the peripheral nervous system or muscle. (A) Thermogenetic activation revealed that some lines were sleep promoting and others wake promoting. (B-F) MIP images of GFP-driven expression in the brain and VNC of the four spilt-GAL4 lines. The brain and VNC are outlined in red. Original confocal stacks can be downloaded from www.janelia.org/split-gal4.. The full genotypes of the driver lines are given there and in Supplementary File 1. Statistical comparisons were made by Kruskal-Wallis and Dunn's post hoc test. Asterisk indicates significance from 0: *p<0.05; **p<0.01; ***p<0.001; ****p<0.0001.

bioRxiv preprint doi: https://doi.org/10.1101/2024.10.21.619448; this version posted October 22, 2024. The copyright holder for this preprint (which was not certified by peer review) is the author/funder, who has granted bioRxiv a license to display the preprint in perpetuity. It is made available under aCC-BY 4.0 International license.

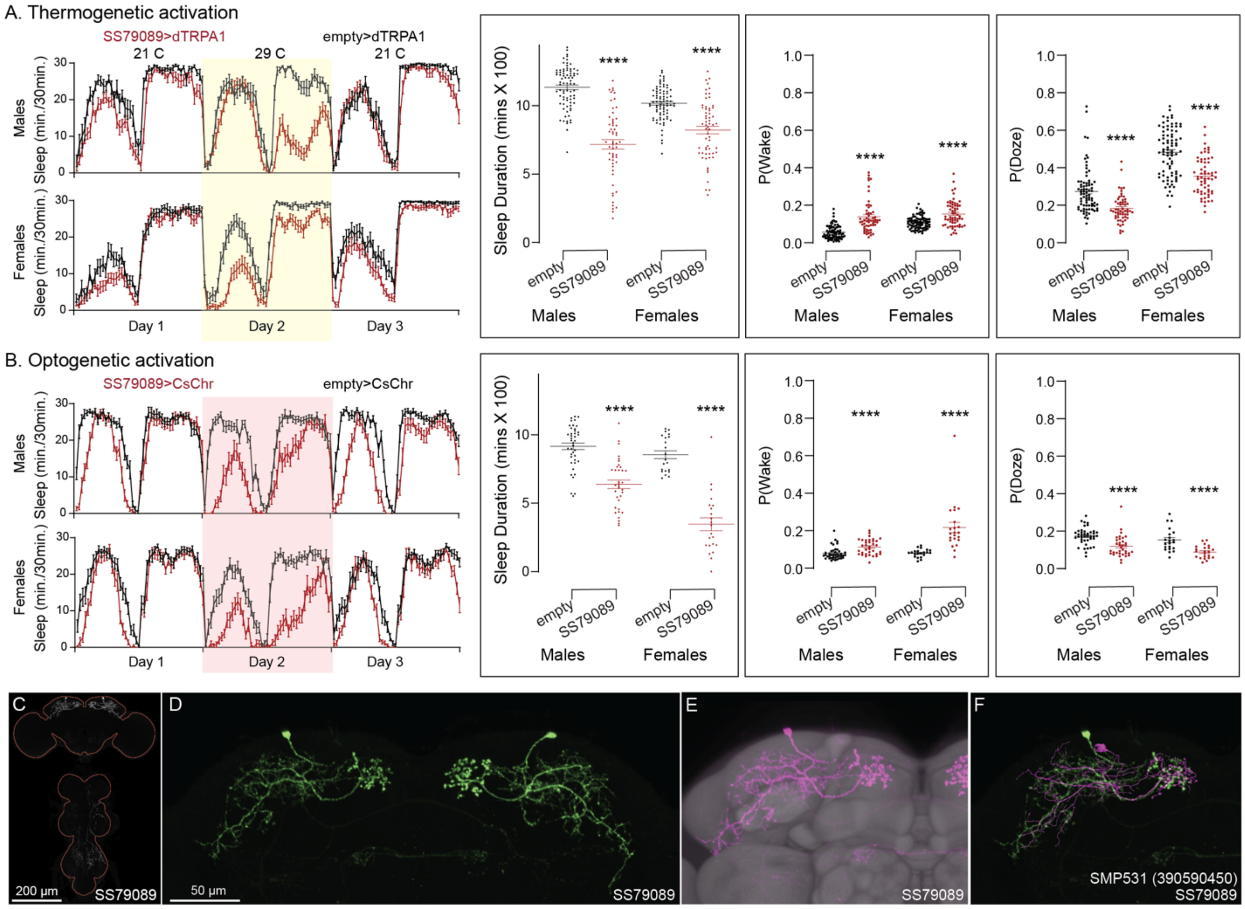


**Figure 11—figure supplement 1**. Activation phenotype of SMP531. SMP531 is part of a potential pathway from clock outputs to the CX (see Figure 12—figure supplement 1) The SMP531 split-GAL4 line SS79089 suppresses sleep when activated thermogenetically (A) and optogenetically (B) in both males and females. Further, activation of this line increases P(Wake) and decreased P(Doze). (C) MIP image of GFP-driven expression in the brain and VNC of SS79089. The brain and VNC are outlined in red. (D) Higher resolution images of the relevant brain area of SS79089. (E) Morphology shown with neuropil reference. (F) Comparison of LM and EM cell morphologies. Original confocal stacks for panels C-E can be downloaded from www.janelia.org/split-gal4. The full genotypes of the driver lines are given there and in Supplementary File 1. Statistical comparisons were made by Kruskal-Wallis and Dunn's post hoc test. Asterisk indicates significance from 0: ****p<0.0001.

bioRxiv preprint doi: https://doi.org/10.1101/2024.10.21.619448; this version posted October 22, 2024. The copyright holder for this preprint (which was not certified by peer review) is the author/funder, who has granted bioRxiv a license to display the preprint in perpetuity. It is made available under aCC-BY 4.0 International license.

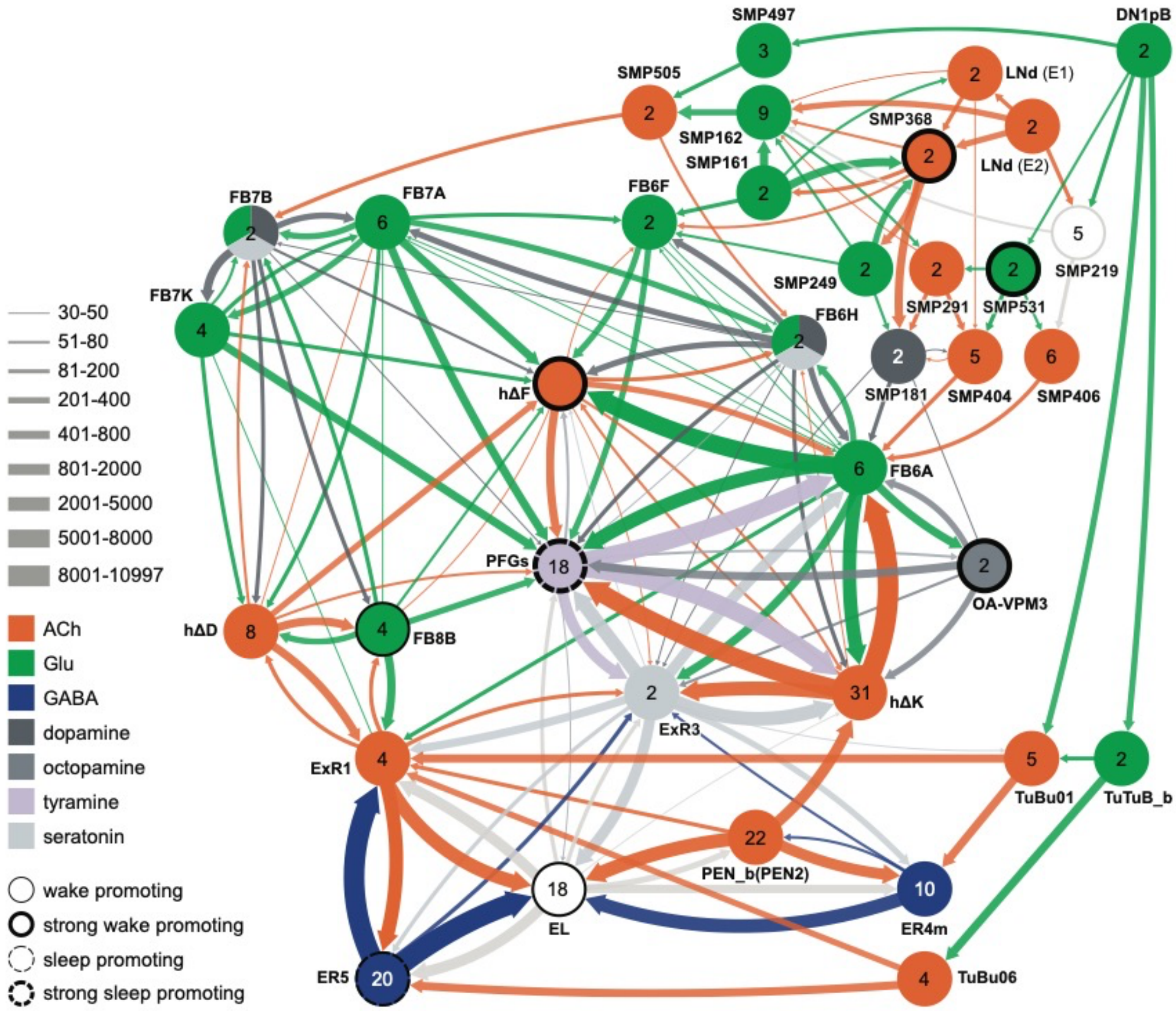


**Figure 12—figure supplement 1**. Circuit diagram showing some additional cell types potentially involved in regulating sleep. Other studies have identified roles for some of these cell types as well as additional cells not shown in this diagram. Links between clock output DN1 neurons to the ExR1 have been described in Lamaze et al. (2018) and Guo et al. (2018). Liang et al. (2019) described a connection form the clock to ExR2 (PPM3) dopaminergic neurons.
